# Effects of dietary supplementation with apple peel powder on the growth, blood and liver parameters, and transcriptome of genetically improved farmed tilapia (GIFT, *Oreochromis niloticus*)

**DOI:** 10.1371/journal.pone.0224995

**Published:** 2019-11-12

**Authors:** Jun Qiang, Omyia Ahmed Mohamed Khamis, Huo Jin Jiang, Zhe Ming Cao, Jie He, Yi Fan Tao, Pao Xu, Jin Wen Bao

**Affiliations:** 1 Key Laboratory of Freshwater Fisheries and Germplasm Resources Utilization, Ministry of Agriculture, Freshwater Fisheries Research Center, Chinese Academy of Fishery Sciences, Wuxi, Jiangsu, China; 2 Beijing Yujing Biotechnology Co., Ltd., Beijing, China; University of Illinois, UNITED STATES

## Abstract

High-density aquaculture and nutritional imbalances may promote fatty liver in genetically improved farmed tilapia (GIFT, *Oreochromis niloticus*), thus reducing the gains achieved by breeding. In this study, apple peel powder (APP) was used as a feed additive for GIFT. A control group (fed on a diet without APP) and five groups fed on diets supplemented with APP (at 0.05%, 0.1%, 0.2%, 0.4%, or 0.8% of the diet, by weight) were established to investigate the effects of APP on GIFT growth performance and physiological parameters, and on gene expression as determined by transcriptomic analysis. Dietary supplementation with APP at 0.2% promoted GIFT growth, reduced total cholesterol and triacylglycerol levels in the serum and liver, and decreased alanine aminotransferase and aspartate aminotransferase activities in the serum. Gene expression profiles in the liver were compared among the control, 0.2% APP, and 0.8% APP groups, and differentially expressed genes among these groups were identified. Annotation analyses using tools at the Gene Ontology and Kyoto Encyclopedia of Genes and Genomes databases showed that the differentially expressed genes were mainly involved in the regulation of immunity and fat metabolism. The results showed that excessive supplementation with APP in the diet significantly inhibited the expression of insulin-like growth factor 2 and liver-type fatty acid-binding protein, and stimulated the expression of fatty acid desaturase 2, heat shock protein 90 beta family member 1, and nuclear factor kappa B. This resulted in disordered lipid metabolism and increased pro-inflammatory reactions, which in turn caused liver damage. Therefore, APP has good potential as an environmentally friendly feed additive for GIFT at levels of 0.1%–0.2% in the diet, but excessive amounts can have adverse effects.

## Introduction

The use of immunostimulants in aquafeeds not only improves the defense responses of fish when they are exposed to pathogens, but also provides an alternative to antibiotics and chemotherapeutics for treating fish diseases [1–4]. The transformation of a raw material into a foodstuff creates various waste streams, which can include edible food mass that is lost, discarded, or degraded at different stages of the food supply chain. Waste streams can include the peel, stems, cores, and skin of fruits, and seeds, husks, bran, and straw from cereals. Food waste has traditionally been viewed as an undesirable material to be disposed of, at considerable expense, via landfill or incineration, or used as animal feed. However, such wastes are now increasingly considered to be a promising source of valuable nutraceuticals [5,6].

Apple peel is a waste product generated during juice and canned fruit production; however, it could be economically beneficial to use it as a value-added food ingredient [7,8]. Given its high levels of dietary fiber, apple peel can promote lipid metabolism and blood glucose regulation. In previous studies, apple peel extract (APE) was shown to have an inhibitory effect on insulin resistance-related obesity and type II diabetes in mice fed on a high-fat diet [9]. In those mice, dietary supplementation with APE resulted in significantly improved glucose tolerance and insulin sensitivity, reduced cytokine levels in the early phase of pro-inflammation, and decreased oxidation levels in adipose tissue [9]. Li et al. [10] found that hyperlipidic mice fed on a diet supplemented with 4% apple dietary fiber showed significant reductions in blood glucose and blood fat.

Increasing fishmeal prices have led to the development of high-fat diets for farmed fish. A high-fat diet provides sufficient dietary protein for tissue synthesis, increases dietary protein utilization efficiency, and saves protein resources [11]. However, high-fat diets may cause imbalances between pro-inflammatory and anti-inflammatory activities [12]. Pro-inflammatory cytokines, including interleukin (IL)-1β and tumor necrosis factor (TNF)-α, are involved in the inhibition of hepatic fat metabolism in genetically improved farmed tilapia (GIFT, *Oreochromis niloticus*) [12]. Nuclear factor κB (NF-κB) is an important transcription factor that is activated during lipid metabolism disorder in grass carp (*Ctenopharyngodon idella*) [13] and during liver injury in Nile tilapia [14]. Activated NF-κB regulates the expression of several pro-inflammatory and cytotoxic cytokines during inflammation.

Previous studies have shown that the components of red delicious apple peel include triterpenoids, flavonoids, organic acids, and plant sterols [15], and about 80% of total polyphenols are concentrated in the peel of apple fruits [16]. Studies on mammals have shown that triterpenoids and polyphenols can reduce the production of pro-inflammatory cytokines by inhibiting transcription factors and signaling pathways [17,18]. Mueller et al. [17] found that triterpenoids in apple peel exerted anti-inflammatory effects by modulating the expression of the gene encoding IFN-γ-inducible protein-10 in T84 cells; this protein plays an important role in inflammatory bowel disease. The administration of tea polyphenols at a dose greater than 50 mg/kg ameliorated the effects of type II diabetes mellitus in rats, accompanied by lower levels of total cholesterol (TC), triglyceride (TG), free fatty acids (FFA), and low-density-lipoprotein cholesterol (LDL-C) in the blood and reduced levels of inflammatory factors (TNF-α, IL-1β, and IL-6) [18]. The ability of these compounds to inhibit inflammatory factors and affect regulatory signaling pathways suggests that they have the potential to treat and prevent fatty liver inflammation. Therefore, the main focus of this study was to determine whether apple peel can be used as a feed additive to regulate fish lipid metabolism and alleviate inflammatory damage.

GIFT is a freshwater farmed fish with high economic and nutritional value. The liver is an important organ in fish metabolism. Once it is damaged or diseased, metabolic disorders and low disease resistance can develop, which may lead to other secondary diseases [19]. In high-density intensive GIFT aquaculture, feed nutrition is not balanced, especially with the recent trend towards high-fat diets [20]. A high-fat diet can accelerate fish growth, but long-term consumption of such diets can lead to metabolic disorders, fat accumulation, fatty liver, and ultimately death due to liver necrosis or hemorrhage. This can seriously reduce the gains achieved by breeding [20]. Therefore, the development of environmentally friendly feed additives that protect liver function and promote tilapia growth is important for research and industry. For example, supplementation with 100 mg/kg silymarin or 2% chitosan in the diet can increase the growth and feed coefficient of tilapia [21, 22]. Against this background, the main purposes of this study were: (i) to investigate the effect of APP on the fat metabolism and fat deposition in the liver in GIFT; (ii) to determine how this diet affects signal-regulated pathways in the liver by transcriptomic analyses, with a focus on fat metabolism and inflammatory responses; and (iii) to screen for differentially expressed genes between GIFT fed on diets without APP and those with various levels of APP and verify their expression levels by qRT-PCR. The results of this study shed light on the molecular mechanisms of the effect of APP on liver function and fat metabolism and inflammatory responses in GIFT. These results also provide theoretical support for the use of APP as an additive in aquatic feed.

## Materials and methods

### Ethics approval

The study protocols and design were approved by the Ethics Committee at the Freshwater Fisheries Research Centre of the Chinese Academy of Fishery Sciences (Wuxi, China). The GIFT were maintained in well-aerated water and treated with 100 mg/L tricaine methanesulfonate (Sigma, St Louis, MO, USA) for rapid deep anesthesia. All samples were extracted based on the Guide for the Care and Use of Laboratory Animals in China.

### Experimental diets

The experimental diets included a control (no APP) and five supplemented with APP at different concentrations (0.05%, 0.1%, 0.2%, 0.4%, and 0.8% by weight) (Table 1). For this, commercially available APP was provided from Beijing Yujing Biotechnology Co., Ltd. (Beijing, China). All ingredients in the diets were powdered and thoroughly mixed together in a food mixer for about 15 min, after which APP was added to different levels. Each diet was mixed for about 40 min until well homogenized, and then tap water was added to produce a firm dough. The dough was passed through a pelleting machine with 1-mm diameter; and the extruded dough was broken and sieved while fresh to obtain pellets of a convenient size. Each diet was dried at ambient temperature for 3 days and then stored at −20°C in labeled plastic-lined bags until use.

**Table 1:** Ingredients and composition of basal diet.

Ingredients (%)	0 (Control)	0.05	0.1	0.2	0.4	0.8
Fish meal	8	8	8	8	8	8
Wheat middling	15	15	15	15	15	15
Corn starch	16.8	16.8	16.8	16.8	16.8	16.8
Soybean oil	5	5	5	5	5	5
Soybean meal	15	15	15	15	15	15
Cottonseed meal	18	18	18	18	18	18
Rapeseed meal	18	18	18	18	18	18
Vitamin premix^1^	0.5	0.5	0.5	0.5	0.5	0.5
Mineral premix^2^	0.5	0.5	0.5	0.5	0.5	0.5
Choline chloride	0.5	0.5	0.5	0.5	0.5	0.5
Vit C phosphate ester	0.2	0.2	0.2	0.2	0.2	0.2
Ca (H_2_PO_4_)_2_	1.5	1.5	1.5	1.5	1.5	1.5
Cellulose	1	0.95	0.9	0.8	0.6	0.2
Total	100	100	100	100	100	100
*Proximate composition (%*, *DM)*						
Crude protein	28.7	28.2	28.5	28.7	28.5	28.8
Crude lipid	7.3	7.2	7.3	7.4	7.5	7.3

^1^Vitamin premix (mg/kg dry diet): V_A_, 10; V_D_, 0.05; V_E_, 400; V_K_, 40; V_B1_, 50; V_B2_, 200; V_B3_, 500; V_B6_, 50; V_B7_, 5; V_B11_, 15; V_B12_, 0.1; V_C_, 1000; Inositol, 2000; Choline, 5000.

^2^Mineral premix (mg/kg dry diet): FeSO_4_.7H_2_O, 372; CuSO_4_.5H_2_O, 25; ZnSO_4_.7H_2_O, 120; MnSO_4_.H_2_O, 5; MgSO_4_, 2475; NaCl, 1875; KH_2_PO_4_, 1000; Ca (H_2_PO_4_)_2_, 2500.

### Experimental facility and fish rearing

A total of 540 healthy GIFT fingerlings with an average body weight of 2.57 ± 0.03 g were obtained from the Tilapia Breeding Center, Freshwater Fisheries Research Centre, Wuxi, China. Fingerlings were acclimated for 1 week under a natural photoperiod with continuous aeration and a water recirculating system. During this period, the fish were fed three times with a commercial diet (crude protein 31.5%; crude fat 7.5%). Subsequently, they were randomly divided into six groups. Each group was fed on the experimental diet until apparent satiation three times a day (08:00, 12:00, and 16:00) for 8 weeks. Each group had three replicates or plastic tanks (30 fish per tank, 0.9 × 0.9× 1.0 m). Water temperature (29.0°C ± 0.5°C) and pH (pH = 7.4) were kept constant during the experimental period. Water quality was checked once a week. The ammonia, nitrate, and nitrite levels were all <0.1 mg/L, and dissolved oxygen was maintained at 5.94 ± 0.26 mg/L.

### Sample collection and fish growth performance

At 24 h after the last experimental feeding, the body weight of all of the fish in each tank was measured. For each group, nine fish (three fish per tank) were weighed and dissected, and their liver and viscera were removed and separately weighed. Fish growth was assessed in terms of weight gain (WG), specific growth rate (SGR), hepatosomatic index (HSI), and viscerosomatic index (VSI). Feed utilization was analyzed using feed conversion ratio (FCR). Throughout the experiment, the amount of feed consumed and mortality in each replicate were noted. These parameters were calculated as follows:

$$WG(g)=W_{2}-W_{1}$$

$$SGR(\%{\mathrm{day}}^{-1})=[Ln(W_{2})-Ln(W_{1})/t]\times 100$$

$$HSI(\%)=[L{\mathrm{iver}\;\mathrm{weight}(g)/W}_{2}(g)]\times 100$$

$$VSI(\%)=[V{\mathrm{isceral}\;\mathrm{weight}(g)/W}_{2}(g)]\times 100$$

FCR=Feedintake(g)/WG(g)

Survival(%)=[Numberofsurvivingfish/initialnumberoffish]×100

where *W*_2_ is final weight (g), *W*_1_ is initial weight (g), and t is the feeding trial period (days).

### Blood and hepatic parameters

The blood of GIFT was analyzed to determine hematological and biochemical parameters. At 24 h after the last feeding, blood was collected from the caudal vein of four anesthetized fish per tank. Each blood sample was transferred into an Eppendorf tube, left to clot at 4°C, and then centrifuged at 3500 g and 4°C for 10 min. The collected serum was then stored at −20°C until further biochemical analysis (within 10 h). Serum glucose (GLU), TC, and TG levels and serum alanine aminotransferase (ALT) and aspartate aminotransferase (AST) activities were quantified by an electrochemiluminescence method using a Mindray biochemical auto analyzer (BS-400), with kits supplied by Mindray Biomedical Electronics Co., Ltd. (Shenzhen, China) [23]. The hepatic TG, TC, and FFA levels were measured using detection kits purchased from Nanjing Jiancheng Biological Engineering Research Institute (Nanjing, China) [24].

### Liver samples for high-throughput sequencing

On the basis of data on growth and related biochemical indicators, an appropriate APP group (APP_A), a high-dose APP group (APP_H), and a control group (C) were further analyzed. Five fish (45 fish in total) were taken from each tank and their liver tissues were dissected. The liver tissue of each fish was divided into two parts, which were immediately frozen in liquid nitrogen and stored at −80°C until use. One part was used for transcriptome sequencing and the other was used for experiments to confirm the transcript levels of differentially expressed (DE) genes identified from the sequencing data.

### Analysis of transcriptome libraries

#### RNA extraction and transcriptome sequencing

The stored liver samples were used for transcriptome sequencing. Total RNA was extracted using Trizol reagent (Invitrogen, CA, USA) following the manufacturer’s protocol, and RNA quantity and purity were analyzed using a Bioanalyzer 2100 and RNA 6000 Nano LabChip Kit (Agilent, CA, USA) with RNA Integrity Number >7.0 and 28S/18S ≥1.5. The RNA samples with good integrity and purity for each group were mixed, and five fish per tank were combined as a treatment group to construct each mRNA library. About 10 μg total RNA from each mixed group was used to prepare libraries. Nine mRNA libraries were constructed, namely, APP_A1, APP_A2, APP_A3, APP_H1, APP_H2, APP_H3, C1, C2, and C3. The experimental procedures, including those for library preparation and sequencing, were the standard procedures provided by Illumina [25, 26]. Each library was qualified and sequenced using the Illumina Hiseq4000 platform. The sequences were double-ended 2 × 150 bp (PE150) reads.

#### Assembly and annotation of transcripts

Invalid reads (including joint, repetitive, and low-sampling reads) were filtered from the sequence data to obtain clean reads, which were used for subsequent analyses. The data processing steps to obtain clean reads were as follows: 1) remove reads containing adaptors; 2) remove reads with N >5%; and 3) remove low-quality reads (those with more than 20% of bases with a mass value Q≤10). The short-read assembly software Trinity 2.4.0 (Campton, NH, USA) (http://trinityrnaseq.sourceforge.net/) was used for *de novo* transcriptome reconstruction. The raw sequence data have been submitted to NCBI’s Gene Expression Omnibus and are accessible under the GEO series accession number GSE127810 (https://www.ncbi.nlm.nih.gov/geo/query/acc.cgi?acc=GSE127810). Reads from the APP_A, APP_H, and C libraries were aligned to the *Oreochromis niloticus* reference genome (http://asia.ensembl.org/Oreochromisniloticus/Info/Index/dna/) using the TopHat 2 package, which initially removes a portion of the reads based on quality information accompanying each read and then maps the reads to the reference genome [27]. TopHat, which allows multiple alignments per read (up to 20 by default) and a maximum of two mismatches when mapping the reads to the reference, was used to build a database of potential splice junctions and confirm them by comparing previously unmapped reads against the database of putative junctions.

#### Enrichment analysis of differentially expressed genes

In transcriptome RNA-seq data, the gene expression level is represented by fragments per kilobase of exon model per million mapped reads (FPKM) values [28]. The Cuffdiff command of Cufflinks 2.2.1 software (http://cufflinks.cbcb.umd.edu/index.html) was used to identify the DE genes among libraries [29]. In this study, the false discovery rate (FDR) was calculated using corrected *P*-values. The DE genes between samples were selected based on |log2foldchange|≥1 and corrected *P*<0.05. Gene Ontology (GO) and Kyoto Encyclopedia of Genes and Genomes (KEGG) enrichment analyses were performed to determine the functions of the DE genes and the metabolic pathways associated with them, respectively.

#### Gene Ontology analysis of differentially expressed genes

Gene Ontology is an internationally standardized gene function classification system that provides a dynamically updated and controlled vocabulary to fully describe the properties of genes and gene products in organisms. There are three ontological groups: molecular functions, cellular components, and biological processes. The basic GO unit is a “term,” and each term corresponds to an “attribute.” Significant enrichment analysis of GO function first maps all significant DE genes to the terms in the GO database (http://www.geneontology.org/), then calculates the number of genes per term, and then applies a hypergeometric test to find GO entries that are significantly enriched in DE genes compared with the entire genomic background. The calculation is as follows:

$$P=1‐\sum_{i=0}^{m-1}\frac{\left(\frac{M}{i})(\frac{N-M}{m-i}\right)}{\left(\frac{N}{n}\right)}$$

where *N* is the total number of genes with GO annotations, *n* is the number of DE genes in *N*, *M* is the number of genes annotated as a particular GO term, and *m* is the number of DE genes annotated as a particular GO term.

#### KEGG enrichment analysis of DE genes

Different gene products coordinate with each other *in vivo* to perform biological functions; hence, pathway-based analyses can shed light on the biological functions of genes. The tools at the KEGG database (http://www.genome.jp/kegg/pathway.html) can be used to identify pathways significantly enriched with DE genes. Hypergeometric testing is used to identify pathways significantly enriched with DE genes compared with the entire genome background. The calculation is as follows:

$$P=1‐\sum_{i=0}^{m-1}\frac{\left(\frac{M}{i})(\frac{N-M}{m-i}\right)}{\left(\frac{N}{n}\right)}$$

Here, *N* is the total number of genes with KEGG annotations, *n* is the number of DE genes in *N*, *M* is the number of genes annotated with a particular pathway, and *m* is the number of DE genes annotated with a particular pathway.

#### Verification of DE genes by qRT-PCR

All primers for screened DE genes (Table 2) were synthesized by the Shanghai Jikang Biotechnology Co., Ltd. (Shanghai, China). Another liver sample, obtained as described in section 2.5, was selected for RNA extraction. The RT reaction and qRT-PCR were carried out in accordance with the instructions of PrimeScript RT Reagent Kit Perfect Real Time (TaKaRa, Dalian, China) and SYBR^®^ Premix Ex Taq (TaKaRa) kits, respectively, using the ABI 7900HT Fast Real-Time PCR System. The internal reference was 18S rRNA. The PCR mixture (50 μL) consisted of 19 μL autoclaved deionized water, 25 μL SYBR Green PCR Master Mix (2×), 2 μL forward and reverse primers (10 mol/L), and 4 μL cDNA working solution. The cycling conditions were as follows: 95°C for 5 min, 95°C for 15 s, and 60°C for 60 s (40 cycles), with a final dissociation step at 95°C for 15 s, 60°C for 15 s, and 95°C for 15 s [19]. Three replicate wells were used for each reaction. All test samples contained a negative control without a template to rule out false positive results.

**Table 2:** Sequences of primers used for qRT-PCR.

Name		Primer sequence (5′–3′)
LPL	F: 5′- ATCAGCACTACCCGACCTCT-3′ R: 5′- GCGCTCCCAGACTATAACCC-3′	
IGF-2	F: 5′- CGCCTAACTCACCTGCAATC-3′ R: 5′- TGTCCGTATCTTTGCTGGGT-3′	
FABP2	F: 5′-TTCGAAGACATCCACGCAGT-3′ R: 5′-AGTTTTGGGAGGCTGTCACT-3′	
TLR2	F: 5′- CGCACAGATAAGGCAGACAC-3′ R: 5′- CCTAGTCCCAGAGCTGCTTT-3′	
HSP90b1	F: 5′- AGACTACCTGGAGCTGGAGA-3′ R: 5′- TCAACCGTCTCAGTCTTGCT-3′	
FADS2	F: 5′- GCAGGAATGATCAGTGGCTG-3′ R: 5′- CTCCGTAGCATCCTCTCCAG-3′	
NF-kB	F: 5′- AACCCCATCTACGACAGCAA-3′ R: 5′- TCCCAGCATCCATCCTCATC-3′	
18S rRNA	F: 5′-GGCCGTTCTTAGTTGGTGGA-3′ R: 5′-TTGCTCAATCTCGTGTGGCT-3′	

LPL: lipoprotein lipase; IGF-2: insulin-like growth factor 2; FABP2: liver-type fatty acid-binding protein; TLR2: toll-like receptor 2; HSP90b1: heat shock protein 90 beta family member 1; FADS2: fatty acid desaturase 2; NF-kB: nuclear factor kappa B

### Hematoxylin–eosin staining and oil red O staining of frozen sections

#### Hematoxylin–eosin staining [20]

Three fish were randomly selected from each tank, their liver tissue was removed and then fixed with 4% paraformaldehyde for 24 h. After washing with tap water, the liver tissue was subjected to gradient alcohol dehydration, cleared with xylene, and then embedded in paraffin embedding. Slices (5 μm) were cut using an RM-2145 (Leica, Nussloch, Germany) rotary microtome. After hematoxylin–eosin (HE) staining, sections were observed under a Leica UB203I optical microscope and photographed.

#### Oil red O staining

Oil red O staining was conducted as described elsewhere [20]. Briefly, liver tissues were fixed with 4% paraformaldehyde for 4 days, washed twice with phosphate buffer, washed twice with phosphate-buffered solution, immersed in 30% sucrose (prepared in phosphate buffer) at 4°C overnight, embedded in OCT (ice cutting embedding agent), and cut into 8-μm sections with using a Leica CM1950 cryostat. The sections were heated at 60°C for 30 min, stained for 10 s with hematoxylin staining solution, washed for 1 min tap water, dried, rinsed in 50% ethanol, stained with oil red O ethanol dye solution for 8 min, washed with 50% ethanol and then tap water, and then sealed with glycerin gelatin.

### Data analysis

The relative expression level of each gene in each APP dietary supplementation treatment *vs*. the control was determined by the 2^−ΔΔCT^ method. The results are expressed as mean ± standard deviation (SD). First, the data were tested for a normal distribution and homogeneity of variance; then, an appropriate analysis was selected depending on the results. The relationship between SGR and dietary APP levels was analyzed using a two-slope broken-line model. The significance level was set at *P* < 0.05.

## Results

### Growth performance and feed utilization efficiency

**Fig 1:**
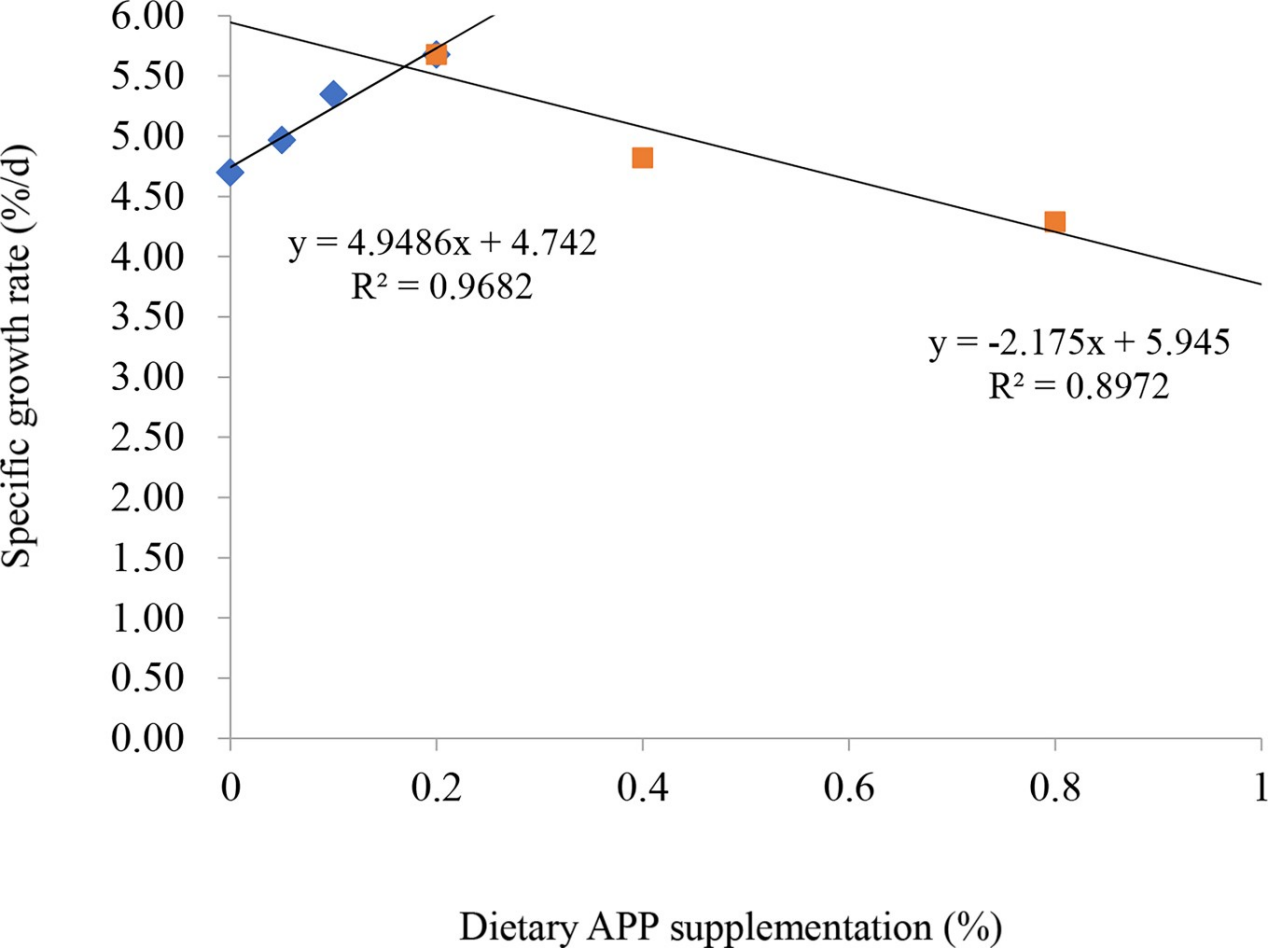
Relationship between specific growth rate and amount of apple peel powder (APP) in feed, as analyzed by two slope broken-line model.

The WG and SGR were significantly lower in 0.4%–0.8% APP groups than in the control group and the 0.05%–0.2% APP groups (*P* <0.05) (Table 3); however, there was no significant difference in WG or SGR between the control group and the 0.05%–0.2% APP groups. The FCR was higher in the 0.8% APP group and in the control than in the 0.2% APP group. The broken-line regression model of SGR indicated that 0.17% APP was the optimal amount for GIFT to achieve optimal growth (Fig 1). The HSI and VSI were significantly lower in the 0.2%, 0.4%, and 0.8% APP groups than in the control group. However, there were no significant differences in HSI and VSI among the control and the 0.05% and 0.1% APP groups (*P* > 0.05). Dietary supplementation with 0%–0.8% APP did not significantly affect survival.

**Table 3:** Growth performance and feed utilization of GIFT (*Oreochromis niloticus*) fed on diets supplemented with apple peel powder for 8 weeks.

Parameters	Dietary apple peel powder level (%)					
	0	0.05	0.1	0.2	0.4	0.8
WG	41.12±2.45^b^	48.38±2.39^ab^	51.94±2.86^a^	54.65±2.92^a^	42.43±2.87^b^	31.46±4.33^c^
SGR(%/d)	4.70±0.25^b^	4.97±0.12^b^	5.34±0.24^ab^	5.68±0.20^a^	4.82±0.12^b^	4.29±0.23^c^
FCR(%)	1.14±0.03^a^	1.05±0.06^ab^	1.04±0.05^ab^	0.92±0.08^c^	1.03±0.04^ab^	1.15±0.04^a^
HSI(%)	1.80±0.08^a^	1.86±0.19^a^	1.74±0.10^ab^	1.64±0.14^b^	1.58±0.06^b^	1.51±0.11^b^
VSI(%)	14.36±0.27^a^	14.27±0.38^a^	13.49±0.19^ab^	12.21±0.27^b^	12.45±0.19^b^	12.81±0.44^b^
Survival (%)	98.89±1.11	100.00±0.00	100.00±0.00	100.00±0.00	96.67±1.93	100.00±0.00

Data were analyzed by one-way analysis of variance and are mean ± standard deviation. Differences among six groups were detected by Duncan’s multiple range test (*P* < 0.05). Different lowercase letters show significant differences among treatment groups.

WG: Weight gain; SGR: Specific growth rate; FCR: Feed conversion ratio; HSI: Hepatosomatic index; VSI: Viscerosomatic index

### Serum biochemical parameters

When the level of APP supplementation in the diet was 0%–0.4%, the serum TG and TC levels of the GIFT significantly decreased with increasing APP (*P* < 0.05) (Table 4). The serum TC and TG contents were significantly lower in the 0.1%, 0.2%, and 0.4% APP groups than in the control group (*P* < 0.05). However, the TC and TG levels were significantly higher in the 0.8% APP group than in the 0.4% group. The serum GLU level was significantly higher in the 0.2% group than in the control group (*P* < 0.05), while there was no significant difference in GLU levels among the 0.05%, 0.1%, 0.2%, 0.4%, and 0.8% APP groups (*P* > 0.05). The serum ALT and AST activities were significantly higher in the 0.8% APP group than in the 0.05%, 0.1%, 0.2%, and 0.4% APP groups.

**Table 4:** Serum biochemistry parameters of GIFT (*Oreochromis niloticus*) fed on diets supplemented with apple peel powder for 8 weeks.

Parameters	Dietary apple peel powder level (%) (*n* = 9)					
	0	0.05	0.1	0.2	0.4	0.8
TC(mmol/L)	4.76±0.52^a^	4.09±0.42^b^	3.65±0.31^bc^	3.51±0.37^bc^	3.12±0.31^c^	3.97±0.41^a^
TG(mmol/L)	5.54±0.64^a^	3.87±0.47^b^	2.69±0.34^c^	2.12±0.31^c^	1.47±0.38^d^	2.38±0.49^c^
GLU(mmol/L)	1.89±0.32^c^	2.17±0.25^ab^	2.14±0.19^ab^	2.33±0.31^a^	2.27±0.17^ab^	2.11±0.30^b^
ALT(U/L)	29.76±1.47^ab^	25.13±2.82^b^	26.13±2.16^b^	26.34±1.92^b^	27.11±2.28^b^	33.24±2.89^a^
AST(U/L)	78.41±6.79^a^	65.41±8.11^b^	72.19±7.33^b^	68.26±5.18^b^	67.22±7.31^b^	84.06±8.23^a^

Data were analyzed by one-way analysis of variance and are mean ± standard deviation. Differences among six groups were detected using Duncan's multiple range test (*P* < 0.05). Different lowercase letters show significant differences among treatment groups.

TC, total cholesterol; TG, triacylglycerol; GLU, Glucose; ALT: alanine aminotransferase; AST: aspartate aminotransferase

### Hepatic biochemical parameters

The levels of TC, TG, and FFA in the liver were significantly lower in the 0.2% APP group than in the control group (*P* < 0.05) (Table 5); however, the levels of hepatic TG and FFA were significantly higher in the 0.8% APP group than in the 0.2% APP group (*P* < 0.05).

**Table 5:** Hepatic biochemical parameters of GIFT (*Oreochromis niloticus*) fed on diets supplemented with apple peel powder for 8 weeks.

Parameters	Dietary apple peel powder level (%) (*n =* 9)					
	0	0.05	0.1	0.2	0.4	0.8
TC(mmol/L)	7.31±0.49^a^	5.19±0.40^b^	4.85±0.41^b^	3.41±0.37^c^	3.82±0.41^c^	3.67±0.38^c^
TG(mmol/L)	21.34±0.64^a^	16.78±0.72^b^	15.79±1.04^bc^	14.12±0.91^c^	16.27±0.68^bc^	17.78±1.49^ab^
FFA(ng/mL)	376.66±16.11^a^	269.40±38.24^b^	262.45±40.55^b^	250.26±34.33^b^	285.47±22.88^b^	361.19±27.54^a^

Data were analyzed by one-way analysis of variance and are mean ± standard deviation. Differences among six groups were detected using Duncan's multiple range test (*P* < 0.05). Different lowercase letters show significant differences among treatment groups.

TC, total cholesterol; TG, triacylglycerol; FFA, free fatty acids

By comparing growth and biochemical parameters among the different groups, we found that the 0.2% APP group of GIFT showed a good growth rate and FCR, with low fat deposition in the liver and serum. Therefore, for further analyses, the 0.2% APP group was selected as APP_A, and the 0.8% group displaying obvious growth inhibition was selected as APP_H.

### Liver histology and lipid droplets

**Fig 2:**
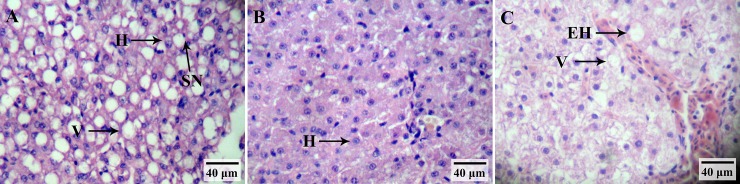
Histological analysis of liver sections by hematoxylin-eosin staining in GIFT (*Oreochromis niloticus*) fed on diets supplemented with 0% (A), 0.2% (B), and 0.8% (C) apple peel powder. H, hepatocyte; V, vacuolization; EH, enlarged hepatocyte; SN, shrunken nucleus.

**Fig 3:**
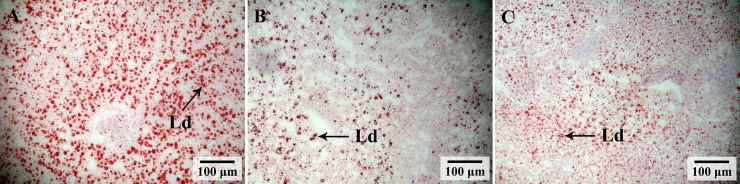
Histological analysis of liver sections by oil red O staining in GIFT (*Oreochromis niloticus*) fed on diets supplemented with 0% (A), 0.2% (B) and 0.8% (C) apple peel powder. Ld, lipid droplet.

In the GIFT fed a control diet for 60 days, the hepatocytes became swollen and part of the nucleus was shrunken, but the cell structure remained intact. The APP_A group showed a normal histological structure in contrast to the control group, and their hepatocyte membrane and nucleus were clear. The hepatocytes in the APP_H group were significantly enlarged, with a shrunken nucleus and some loss of cell membrane integrity (Fig 2). There were significantly more lipid droplets in hepatic cells of the control group had than in those of the APP_A group and the APP_H group, as revealed by oil red O staining (Fig 3).

### Summary of sequencing data and statistics of transcriptome assembly

The transcriptomes of the liver tissues of the C1, C2, C3, APP_A1, APP_A2, APP_A3, APP_H1, APP_H2, and APP_H3 groups were sequenced using the Illumina Hiseq4000 platform. After removing low-quality sequences, the number of valid reads ranged from 40,430,496 to 62,293,208. The Q20 values of the transcriptome data for the nine subgroups from the three treatment groups were 98.26%–99.23%, while the GC contents were 48%–52% (Table 6). These results showed that the transcriptome sequencing data were of good quality and could be used for subsequent splicing and assembly. In the transcriptome data for the C1, C2, C3, APP_A1, APP_A2, APP_A3, APP_H1, APP_H2, and APP_H3 groups, 31,015,020, 32,605,882, 29,847,539, 27,857,982, 35,474,059, 24,377,649, 31,551,102, 38,937,856, and 31,822,452 reads were mapped to the Nile tilapia genome, respectively (S1 Table). Significantly more reads mapped to exonic regions than to intronic and intergenic regions of the genome (S2 Table).

**Table 6:** Overview of reads for mRNA-seq of GIFT (*Oreochromis niloticus*) and quality filtering.

Sample	Raw Reads	Base	Valid Read	Base	Valid Ratio (reads)	Q20%	Q30%	GC content%
APP_A1	45867590	6.88G	45374664	6.81G	98.93	98.59	90.43	49
APP_A2	57594264	8.64G	57040408	8.56G	99.04	99.03	90.79	48.50
APP_A3	52452746	7.87G	40430496	6.06G	77.08	99.23	91.10	52
C1	49272160	7.39G	48844226	7.33G	99.13	99.08	90.64	48.50
C2	54099584	8.11G	53512740	8.03G	98.92	98.47	89.52	49
C3	49681658	7.45G	49111518	7.37G	98.85	98.68	90.00	49
APP_H1	54547964	8.18G	47660140	7.15G	87.37	98.26	86.80	49.50
APP_H2	63096352	9.46G	62293208	9.34G	98.73	99.13	92.26	48
APP_H3	52005944	7.80G	51294450	7.69G	98.63	98.98	91.33	50.50

APP_A: 0.2% apple peel powder; APP_H: 0.8% apple peel powder; C: control group, 0% apple peel powder

### Screening and functional annotation of differentially expressed genes

**Fig 4:**
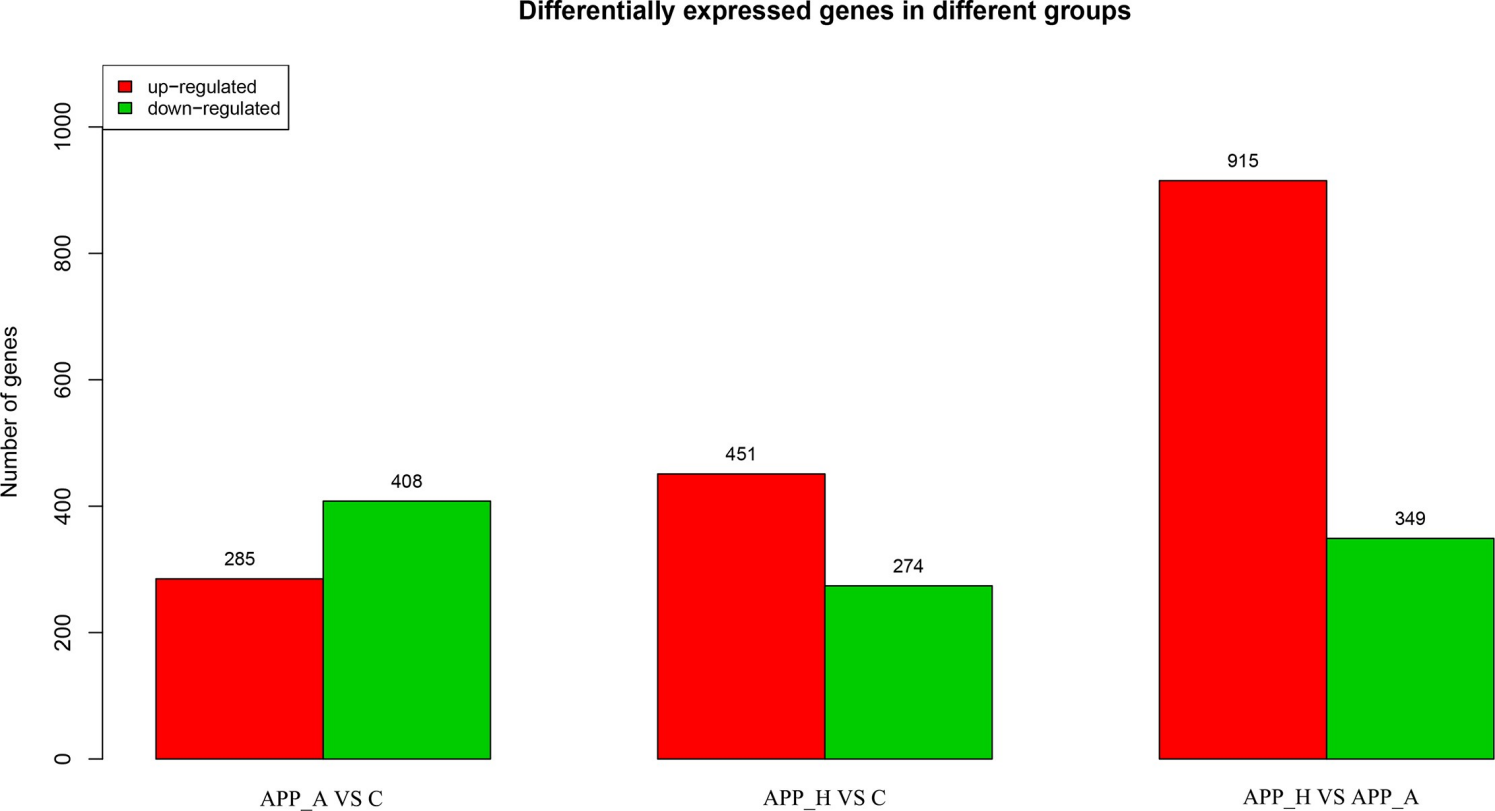
Analysis of differentially expressed genes in APP_A *vs*. C, APP_H *vs*. C, and APP_A *vs*. APP_H group comparisons by transcriptome sequencing. APP_A: 0.2% apple peel powder; APP_H: 0.8% apple peel powder; C: control group, 0% apple peel powder.

**Fig 5:**
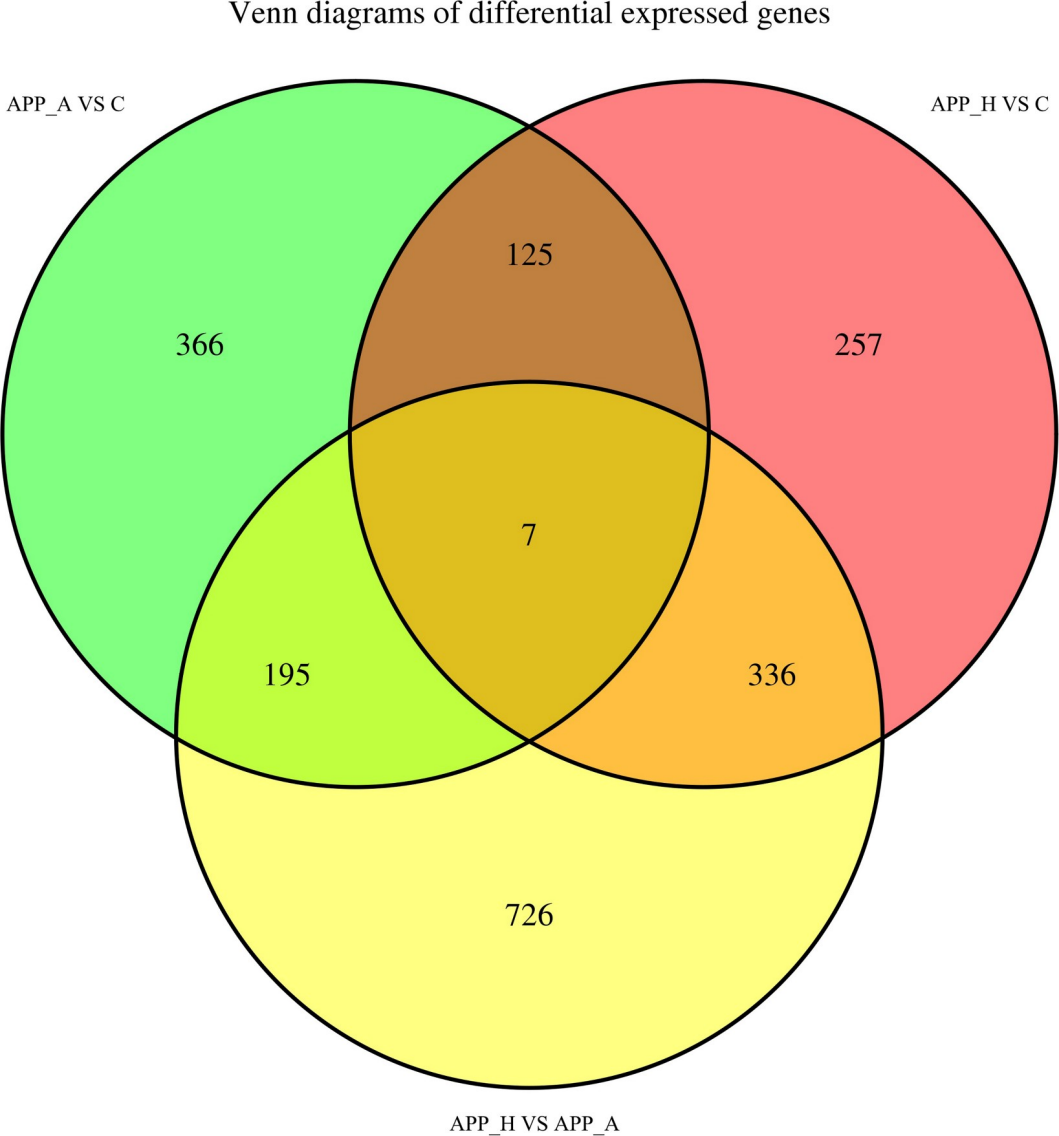
Venn diagram showing differentially expressed genes among APP_A *vs*. C, APP_H *vs*. C, and APP_A *vs*. APP_H group comparisons. APP_A: 0.2% apple peel powder; APP_H: 0.8% apple peel powder; C: control group, 0% apple peel powder.

After normalizing the abundance of genes mapped to the Nile tilapia genome, significantly DE genes were identified on the basis of |log(foldchange)|≥1 and FDR≤0.05. The DE genes are summarized in a histogram and Venn diagram (Figs 4 & 5). Comparisons among the three groups revealed upregulation of 285 DE genes and downregulation of 408 DE genes in the APP_A *vs*. C comparison; upregulation of 451 DE genes and downregulation of 274 DE genes in the APP_H *vs*. C comparison; and upregulation of 915 DE genes and downregulation of 349 DE genes in the APP_A *vs*. APP_H comparison (Fig 4). The number of DE genes specific to the APP_A *vs*. C, APP_H *vs*. C, and APP_A *vs*. APP_H comparisons was 366, 257, and 726, respectively, and seven DE genes were common to all three comparison groups (Fig 5). The seven DE genes encoded lipoprotein lipase (LPL), insulin-like growth factor 2 (IGF-2), liver-type fatty acid-binding protein (FABP2), toll-like receptor 2 (TLR2), heat shock protein 90 beta family member 1(HSP90b1), fatty acid desaturase 2 (FADS2), and nuclear factor kappa B (NF-kB).

### GO functional annotation

The GO functional annotation results showed that dietary supplementation with APP affected the biological processes, cellular components, and molecular functions of tilapia. Compared with the C group, the APP_A group had DE genes in biological processes of fatty acid beta-oxidation, fatty acid biosynthetic process, glycogen metabolic process, and response to virus. The APP_H group had DE genes in biological processes of response to virus, response to stimulus, innate immune response, and inflammatory response (S1 Fig).

### KEGG pathway significant enrichment analysis

**Fig 6:**
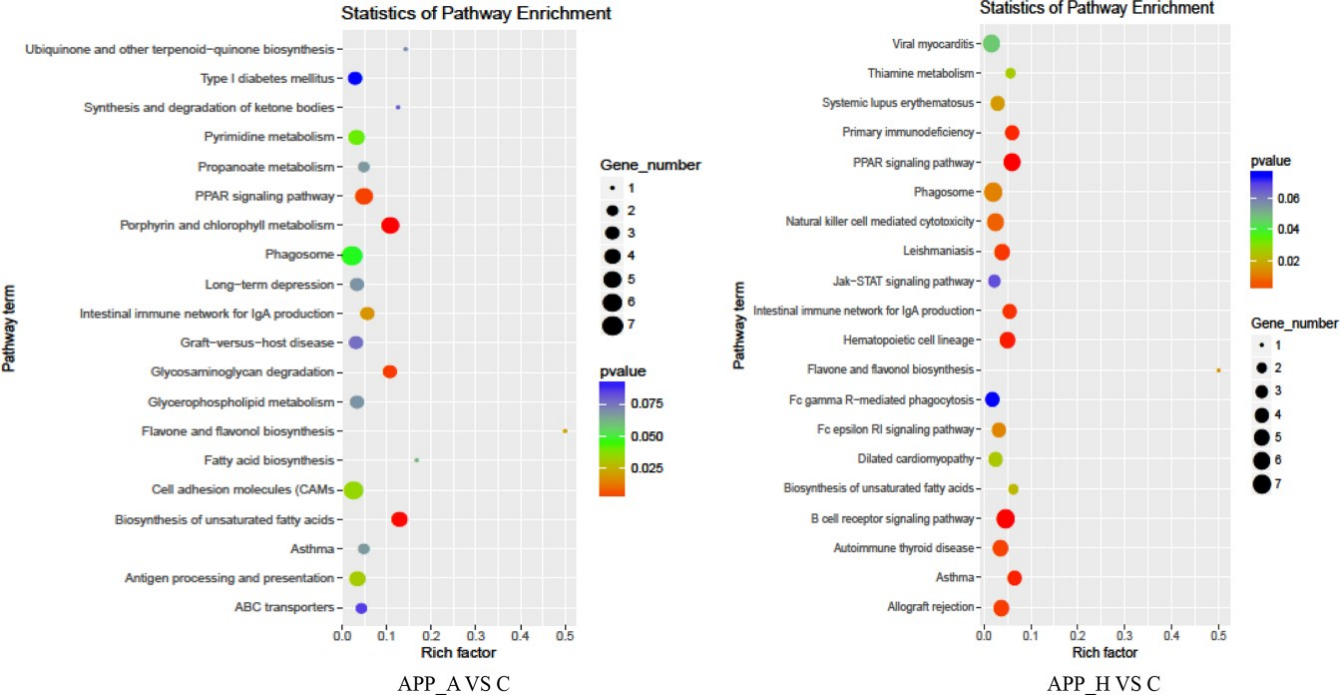
KEGG pathway enrichment analysis of differentially expressed genes (corrected *P*-value < 0.05) in APP_A *vs*. C and APP_H *vs*. C group comparisons. APP_A: 0.2% apple peel powder; APP_H: 0.8% apple peel powder; C: control group, 0% apple peel powder.

The specific signaling pathways enriched with DE genes were identified using tools at the KEGG database. The APP_A group had five pathways with significant enrichment of DE genes compared with the C group: PPAR signaling pathway, phagosome, cell adhesion molecules, biosynthesis of unsaturated fatty acids, and antigen processing and presentation (Fig 6). There were multiple pathways enriched with DE genes in the APP_H group compared with the C group. The top five enriched pathways were viral myocarditis, PPAR signaling pathway, phagosome, natural killer cell-mediated cytotoxicity, and B-cell-receptor signaling pathway. Thus, the pathways enriched in GIFT fed on diets including APP at different levels were mainly involved in the regulation of cellular immunity and fat metabolism.

### Validation of DE genes

**Fig 7:**
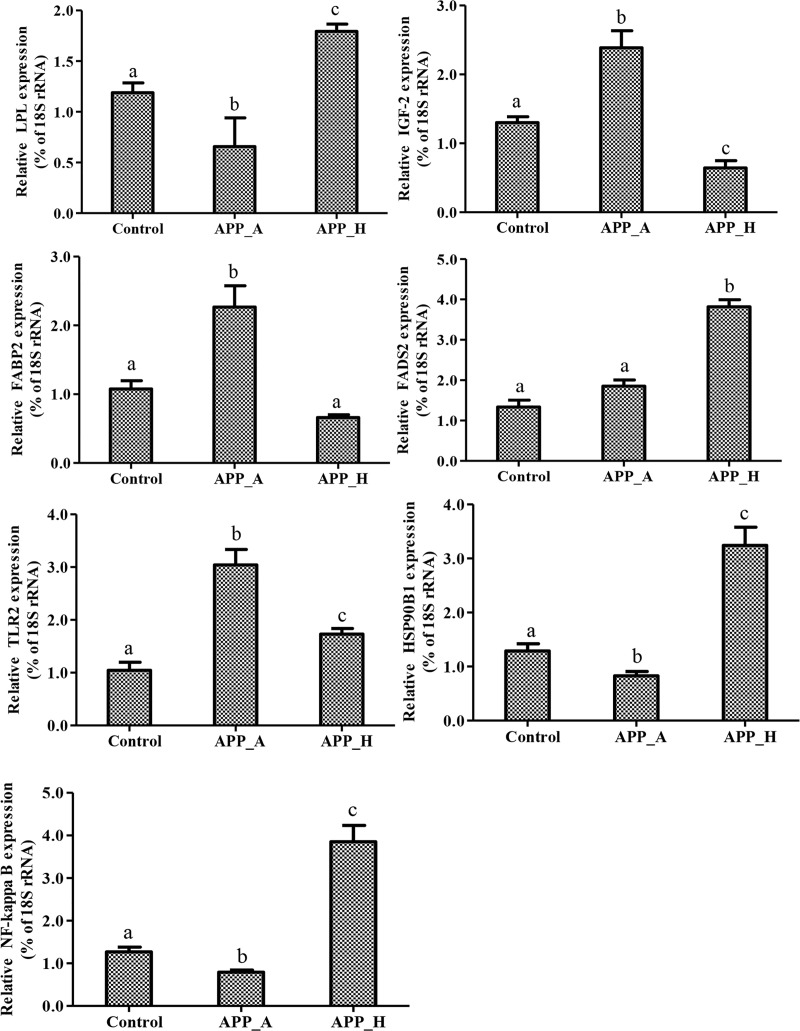
Validation of seven differentially expressed genes in APP_A, APP_H, and C groups by qRT-PCR. Data were analyzed by one-way analysis of variance. Differences between two groups were compared using Duncan's multiple range test (significance at *P* < 0.05). Different lowercase letters show significant differences among different treatment groups. APP_A: 0.2% apple peel powder; APP_H: 0.8% apple peel powder; C: control group, 0% apple peel powder.

The seven DE genes common to all group comparisons were further analyzed by qRT-PCR. The trends in their expression as detected by qRT-PCR (Fig 7) were basically consistent with the RNA-seq results (Table 7). Appropriate supplementation with APP (APP_A group) promoted the expression of *IGF-2*, *FABP2*, and *TLR2* in the liver, and decreased the expression of *LPL*, *HSP90b1*, and *NF-κB*. Excessive supplementation of APP (APP_H group) significantly inhibited the expression of *IGF-2* and *FABP2*, and stimulated the expression of *FADS2*, *HSP90b1*, and *NF-kB*.

**Table 7:** Relative gene expression levels of seven differentially expressed genes in APP_A *vs*. C and APP_H *vs*. C comparisons as estimated from mRNA-Seq data.

Gene abbreviation	Gene description	RNA-seq			
		Log2 (fold_change)	Regulation (APP_A *vs*. C)	Log2 (fold_change)	Regulation (APP_H *vs*. C)
LPL	lipoprotein lipase	-1.67	down	2	up
IGF-2	insulin-like growth factor 2	1.85	up	-2.57	down
FABP2	liver-type fatty acid-binding protein	1.66	up	-2.66	down
TLR2	toll-like receptor 2	3.62	up	1.95	up
HSP90b1	heat shock protein 90 beta family member 1	-1.47	down	2.45	up
FADS2	Fatty acid desaturase 2	1.37	up	3.64	up
NF-κB	nuclear factor κappa B	-1.99	down	2.94	up

APP_A: 0.2% apple peel powder; APP_H: 0.8% apple peel powder; C: control group, 0% apple peel powder

## Discussion

In this study, dietary supplementation with 0.2% APP promoted the growth of GIFT and reduced its FCR. The high dietary fiber content in APP may have had a positive effect on fish health. Studies have shown that fiber supplementation in the diet helps to reduce the energy concentration in food. This allows the body to increase the retention time of food and provides a greater absorption area for nutrients, which are beneficial for other physiological responses that promote growth and development [30]. However, excessive dietary fiber may damage intestinal structure or interact with minerals in the diet, and reducing the efficiency of nitrogen and energy use [31]. This may explain the significantly inhibited growth of GIFT in the 0.8% APP group.

The liver is an important organ for storing fat, and an increase in liver fat content leads to an increase in HSI. In this experiment, compared with GIFT in the C group, those in the 0.2% APP and 0.4% APP groups showed significantly decreased TG and TC levels in liver and serum, and decreased HSI and VSI. These changes may have been related to the functional components of APP. The abundance of dietary fiber in APP can affect metabolism, for example, by stimulating lipid peroxidation and inhibiting fat production in the liver, and by promoting the oxidation of fatty acids in muscles, thereby reducing the accumulation of body fat [32]. The dietary fiber in APP has been shown to reduce plasma sugar and lipid levels in mice under high-fat stress [33]. Foods that are high in fat and cholesterol can increase the concentrations of TC, LDL, and TG in the plasma of mice, and decrease the concentration of high-density lipoprotein (HDL), thereby inducing hypercholesterolemia. However, diseased mice fed dietary fiber from apples showed decreased TC, LDL, and TG levels in plasma, increased HDL, and a reduced risk of cardiovascular disease [33]. The triterpenoids in APP may also alleviate liver fat deposition to some extent. For example, the triterpenoids from *Cyclocarya paliurus* were shown to have a therapeutic effect on nonalcoholic fatty liver disease, by reducing the FFA-induced lipid accumulation in HepG2 cells and reducing the TG content, leading to a decrease in cellular oxidative damage [32]. The activities of serum AST and ALT are indicators of stress. These enzymes showed significantly higher activities in the APP_H group than in the other groups. In addition, the high concentration of FFAs in liver tissue in the APP_H group may have caused severe cytotoxicity, which can lead to hepatocyte damage and the induction of abnormal lipid metabolism [34,35]. This may have contributed to the growth inhibition of GIFT in the APP_H group.

In recent years, transcriptomic analyses have been increasingly used in the fields of immunization, development, and nutrition of aquatic animals. In this experiment, APP_A, APP_H, and C groups were selected for transcriptome analyses to investigate the DE genes and differentially regulated pathways in GIFT fed APP at different levels. Compared with GIFT in the C group, those in the APP_A group had 693 DE genes, of which 285 were upregulated and 408 were downregulated. Seven DE genes were detected in all comparisons among the C, APP_A, and APP_H groups. The results of the GO and KEGG analyses showed that DE genes in APP_A *vs*. C were particularly associated with glucose and lipid metabolism, and immunological regulation; however, the DE genes in APP_H *vs*. C were particularly associated with various immune responses and cell signaling pathways. These findings indicate that abnormal immune stress and signal transduction in the APP_H group may have affected the liver’s metabolic function, leading to inhibition of GIFT growth.

Dietary supplementation with flavonoids or organic acids can help to improve disordered fat metabolism in mice and prevent hyperlipidemia [36,37]. For instance, total flavonoids from *Ligustrum lucidum* were shown to have a beneficial regulatory effect on lipid metabolism disorder in rats in a high-fat model, possibly via regulation of the PPARα-LPL pathway and HMGCR expression [36]. In addition, the total organic acids of *Armeniaca sibirica* were shown to increase the concentration of HDL-C, decrease TG activity, and reduce the serum lipid levels in hyperlipidemic rats, thus reducing blood fat [37]. The flavonoids and organic acids in APP may have played similar roles in GIFT in this study. The liver is an important organ for lipid metabolism in animals, and its lipid metabolic balance is regulated by various factors. The enzyme LPL is mainly synthesized in adipose tissue, the myocardium, and muscle. Its main physiological function is to decompose TG into VLDL, and also to promote the transfer of TC and phospholipids between lipoproteins [38]. Increased LPL activity can affect the levels of serum TC and TG [38]. The higher *LPL* expression levels in the C and APP_H groups may have been related to increased contents of TG and FFAs in the liver. Increased LPL activity increases the decomposition of TG in chylomicrons and VLDL into FFAs, which are then transported to other tissues via the blood to promote lipid synthesis or supply oxidative energy. The high LPL expression levels in the C group may have increased the deposition of fat droplets in the liver. Interestingly, the levels of TC and TG in serum and liver were significantly lower in the APP_H group than in the C group. Therefore, the high LPL expression level in the APP_H group may have been related to the supply of oxidative energy during the immune response and the alleviation of liver stress injury.

FABP2 is a fatty acid binding protein expressed in the liver. It affects fat transport in cells, fat metabolism, and lipid synthesis, and regulates the metabolism of bile acids and TC by long-chain fatty acid-dependent proteins [39]. In another study, mice with knocked-out *FABP2* showed significantly inhibited synthesis of TC and fat oxidation in the liver during fasting, compared with mice in the control group [40]. In addition, a high-fat diet was shown to upregulate the expression of *FABP2* in mouse liver; FABP2 promoted fatty acid transport and alleviated damage caused by high-fat stress [41]. Some aspects of metabolic regulation may be conserved between mammals and aquatic animals. In this study, APP_A stimulated *FABP2* expression in GIFT liver, increased β-oxidation activity and the content of unsaturated fatty acids, and helped to improve the immune response and reduce fat deposition. However, the expression level of *FABP2* in GIFT liver was significantly reduced in the APP_H group, which may have been indicative of liver damage, toxic effects of FFAs, and reduced transport of FFAs from the liver [42].

Polyunsaturated fatty acids (PUFAs) in aquatic animals can be further converted into long-chain PUFAs by a series of dehydrogenation and prolongation reactions catalyzed by fatty acid desaturase (Fad) and elongase [43]. As important membrane components, long-chain PUFAs maintain the fluidity of the cell membrane, thus ensuring the normal physiological function of cells. They can also esterify TC and reduce the TC and TG levels in blood [44]. In our study, the APP_A group showed increased expression of *FADS2*; this may have helped to protect the integrity of the cell membrane and reduce fat deposition in serum. The integrity of liver cell membranes was impaired in the APP_H group. In this group, therefore, elevated *FADS2* expression was insufficient to maintain cell membrane integrity.

Adipose tissue is not only a passive tissue involved in fuel storage and tissue organ filling, but also a huge endocrine system. It secretes a variety of factors that are actively involved in regulating the neuro-endocrine-immune network. Abnormal fat cell differentiation can cause excessive fat accumulation, which in turn leads to endocrine dysfunction of fat cells, and causes the development of insulin resistance and type II diabetes [45]. Recent studies have found that IGF-2 is closely related to diseases of glucose metabolism and fat metabolism in mammals. The overexpression of IGF-2 has been shown to increase incidence of obesity and diabetes [46]. Other studies found that the concentration of IGF-2 was positively correlated with the body weight of pigs during growth, and that increasing concentrations of IGF-2 in plasma were related to increased thickness of backfat [47]. Moreover, when IGF-2 was injected into 4-week-old broilers (0.5 mg/kg), it directly or indirectly caused a change in plasma T3 content and affect the deposition of abdominal fat [48]. In addition, male rats fed a diet supplemented with soy isoflavones showed increased blood IGF activity, which promoted the utilization and decomposition of fat and induced protein synthesis [49]. In this experiment, flavonoids in APP may have stimulated IGF-2 expression in the APP_A group. High levels of IGF-2 may increase fatty acid synthesis and fat storage, promote protein synthesis, and accelerate fish growth. However, the APP_H group showed abnormal metabolism and glucose and fat synthesis, as well as disordered glucose/fat utilization, alongside decreased *IGF-2* expression in liver tissue.

The TLR can initiate innate immunity by recognizing pathogens, and can initiate acquired immunity through signaling. Thus, TLR plays an important role in the body’s immune defense [14]. Flavonoids exert immunomodulatory effects by regulating TLR signaling pathways, particularly the expression of TLR2 and TLR4. Baicalin and quercetin are rich in flavonoids. Baicalin has been shown to reduce the expression of TLR2, TLR4, and MyD88 proteins in rats with renal ischemia–reperfusion injury [50]. Quercetin was shown to reduce the expression of high mobility group box 1 (HMGB1) and the mRNA and protein levels of TLR2 and TLR4 in liver tissues of mice with hepatitis [51]. The flavonoids in APP may have increased the expression level of *TLR2* to stimulate the immune defense response in GIFT. However, the high FFA content in the APP_H group may have acted synergistically with TLR2, inducing an inflammatory response [52].

The GO and KEGG analyses showed that, in the APP_H group, immune and inflammatory response pathways were affected in the GIFT liver. NF-κB is a key factor regulating the transcription of cellular genes. Under normal conditions, it binds to NF-κB inhibitory protein (inhibitor of NF-κB: IκB) and becomes inactive. After stimulation, the IκB kinase (IKK) complex is activated. IκB phosphorylates and dissociates from NF-κB, and the activated NF-κB is transported into the nucleus where it directly initiates and regulates the transcription of genes related to the immune response, cytokines, and adhesion molecules [53]. Flavonoids in baicalin were shown to promote the expression of phosphorylated NF-κB (p-NF-κB) and phosphorylated IκB (p-IκB) proteins in rats with renal ischemia–reperfusion injury, leading to the regulation of immune inflammatory responses [50]. In diabetic mice with myocarditis, the flavonoids in liquiritigenin significantly reduced the secretion of inflammatory cytokines and the phosphorylation level of NF-κB by inhibiting the nuclear factor-κB inhibitor kinase α (IKK-α)/IκB-α signaling pathway [54]. Similar results were found in this study. Our results showed that the flavonoids in APP can protect the liver of GIFT, reduce fat deposition, and inhibit the expression of NF-κB, which may help reduce the secretion of pro-inflammatory cytokines. However, excessive flavonoids may be toxic to the liver. Some flavonoid molecules have similar DNA-embedded molecular structures and are potentially genotoxic and mutagenic [55]. For example, soy isoflavones have been shown to cause or promote DNA oxidative damage in cells of the reproductive system, thereby increasing tumorigenesis [56]. Therefore, the higher flavonoid intake in the APP_H group may have increased liver damage, upregulated the expression of NF-κB, and promoted inflammatory reactions in GIFT.

HSP90, as an important molecular chaperone protein, plays a key role in the development of inflammatory reactions. It has been shown to play an important pro-inflammatory role when retinal pigment epithelial cells develop an inflammatory response caused by non-bacterial infections [57]. In addition, in patients with inflammatory reactions, the expression of HSP32, HSP70, and HSP90 was significantly increased, and the expression of HSP90 was closely related to the inflammatory reaction [58]. However, taurochenodeoxycholic acid was found to inhibit HSP90 gene expression in an arthritis model, exerting an anti-inflammatory effect [59]. Our results showed that the APP_A group had well-functioning liver fat metabolism and good growth, and low levels of inflammatory response signaling factors such as HSP90b1 and NF-κB. A long-term, low-grade pro-inflammatory state of the body is one of the most important factors for obesity and insulin resistance [60]. Although the GIFT in the control group grew normally, the levels of pro-inflammatory factors NF-κB and HSP90b1 in the liver were significantly higher than those in the APP_A group, and may have led to chronic inflammatory reactions, metabolic abnormalities, increased fat deposition, and the development of fatty liver.

## Conclusion

**Fig 8:**
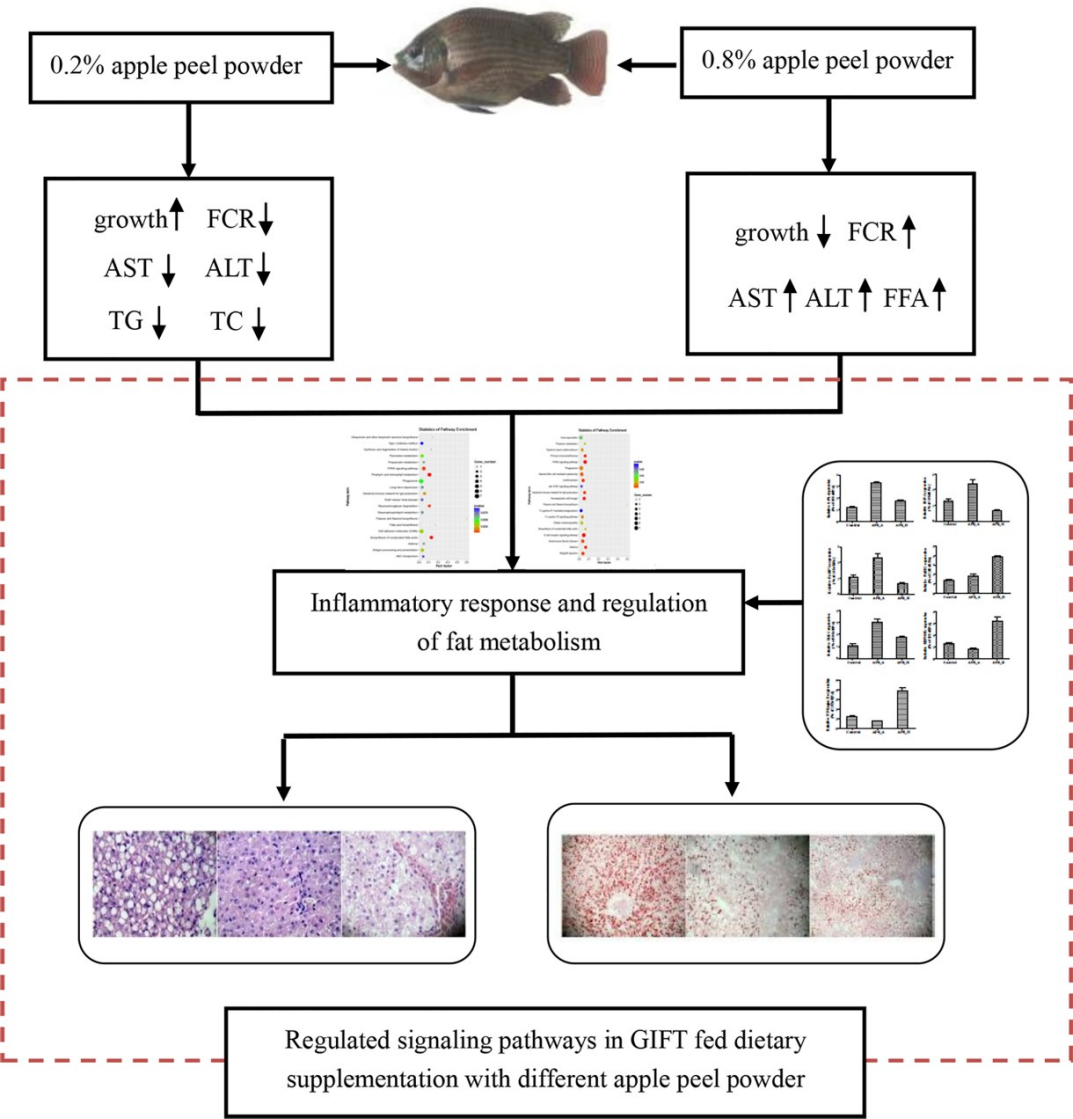
Pathways related to inflammatory response and fat metabolism in GIFT (*Oreochromis niloticus*) potentially affected by apple peel powder in feed.

With the development and use of natural products, apple peel has attracted increasing attention. Its low price and abundance make it a suitable feed additive for aquatic animals. Our results show that appropriate dietary supplementation with APP (APP_A, 0.2%) can promote the growth of GIFT, reduce the levels of TC and TG in the serum and liver, decrease AST and ALT activities in liver tissue, improve lipid metabolism, and inhibit pro-inflammatory reactions (Fig 8). These beneficial effects may be related to the dietary fiber, flavonoids, and organic acids in APP. However, excessive supplementation with APP (APP_H, 0.8%) can lead to disordered lipid metabolism and inflammatory stress (upregulation of HSP90b1 and NF-κB) and increased liver damage. Further research is required to determine how the functional components of APP exert their effects to improve fatty liver, glycolipid metabolism, and inflammatory factors in GIFT.

## Supporting information

S1 Fig GO functional annotation of differentially expressed genes (corrected *P*-value < 0.05) in APP_A *vs*. C and APP_H *vs*. C group comparisons. (DOCX)

S1 Table Summary of read data aligned with *Oreochromis_niloticus* transcriptome. (DOCX)

S2 Table Exonic rates (%) in APP_A, APP_H, and C libraries as determined from mRNA-seq data. (DOCX)
