# Supplementary material for: Effects of dietary supplementation with apple peel powder on the growth, blood and liver parameters, and transcriptome of genetically improved farmed tilapia (GIFT, Oreochromis niloticus)
Source: PLoS One. 2019 Nov 12;14(11):e0224995. doi: 10.1371/journal.pone.0224995 (PMC6850550; doi:10.1371/journal.pone.0224995)
Supplement: S1 Fig — (DOCX) [file pone.0224995.s001.docx]

Effects of dietary supplementation with apple peel powder on the growth, blood and liver parameters, and transcriptome of genetically improved farmed tilapia (GIFT, *Oreochromis niloticus*)

Jun Qiang^1*^, Omyia Ahmed Mohamed Khamis^1^, Huo Jin Jiang^2^, Zhe Ming Cao^1^, Jie He^1^, Yi Fan Tao^1^, Pao Xu^1*^

Jin Wen Bao^1^

1. *Key Laboratory of Freshwater Fisheries and Germplasm Resources Utilization, Ministry ofAgriculture, Freshwater Fisheries Research Center, Chinese Academy of Fishery Sciences, Wuxi214081, Jiangsu, China*

2. *Beijing Yujing Biotechnology Co., Ltd.,Beijing 101100, China*


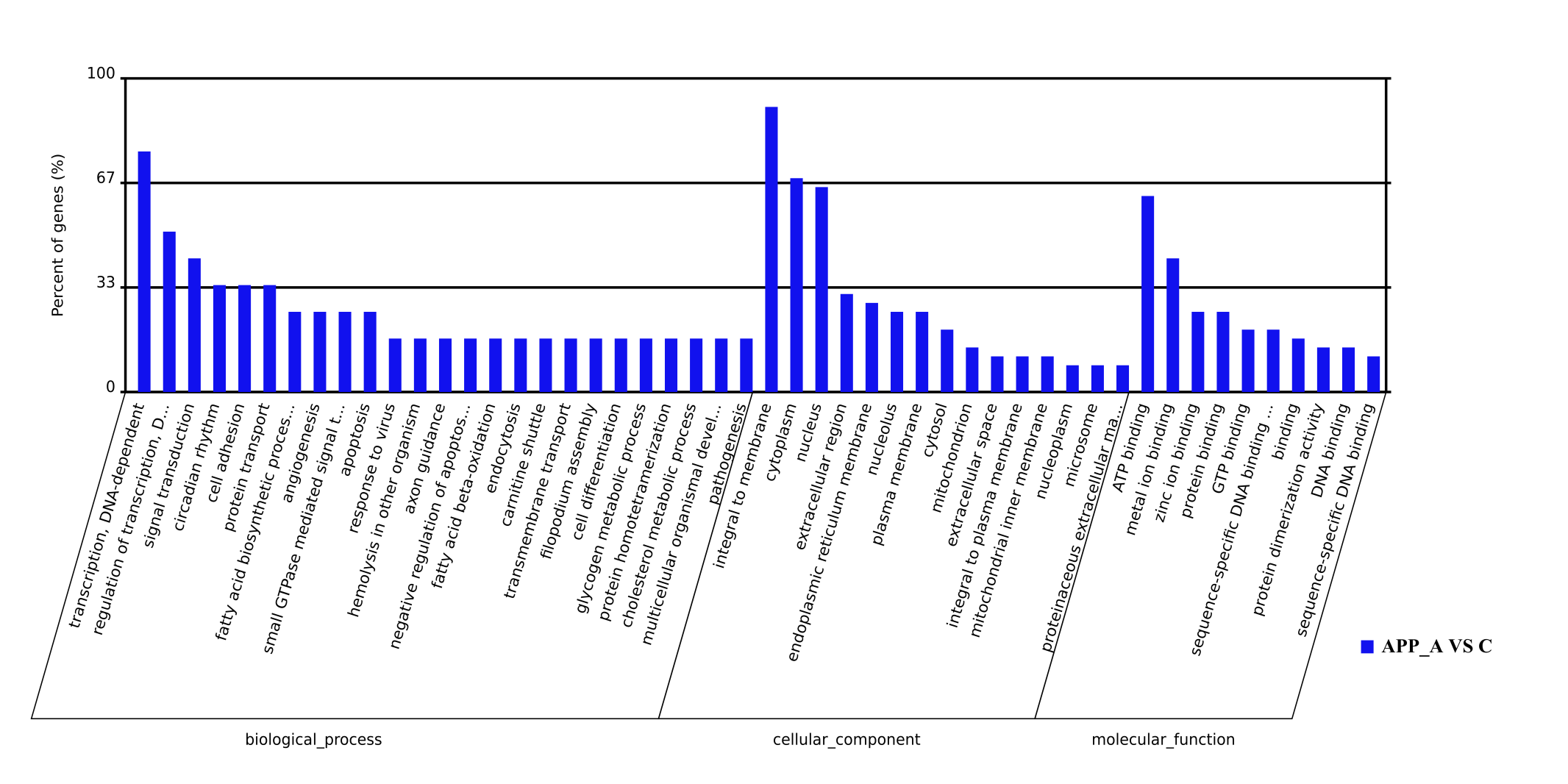


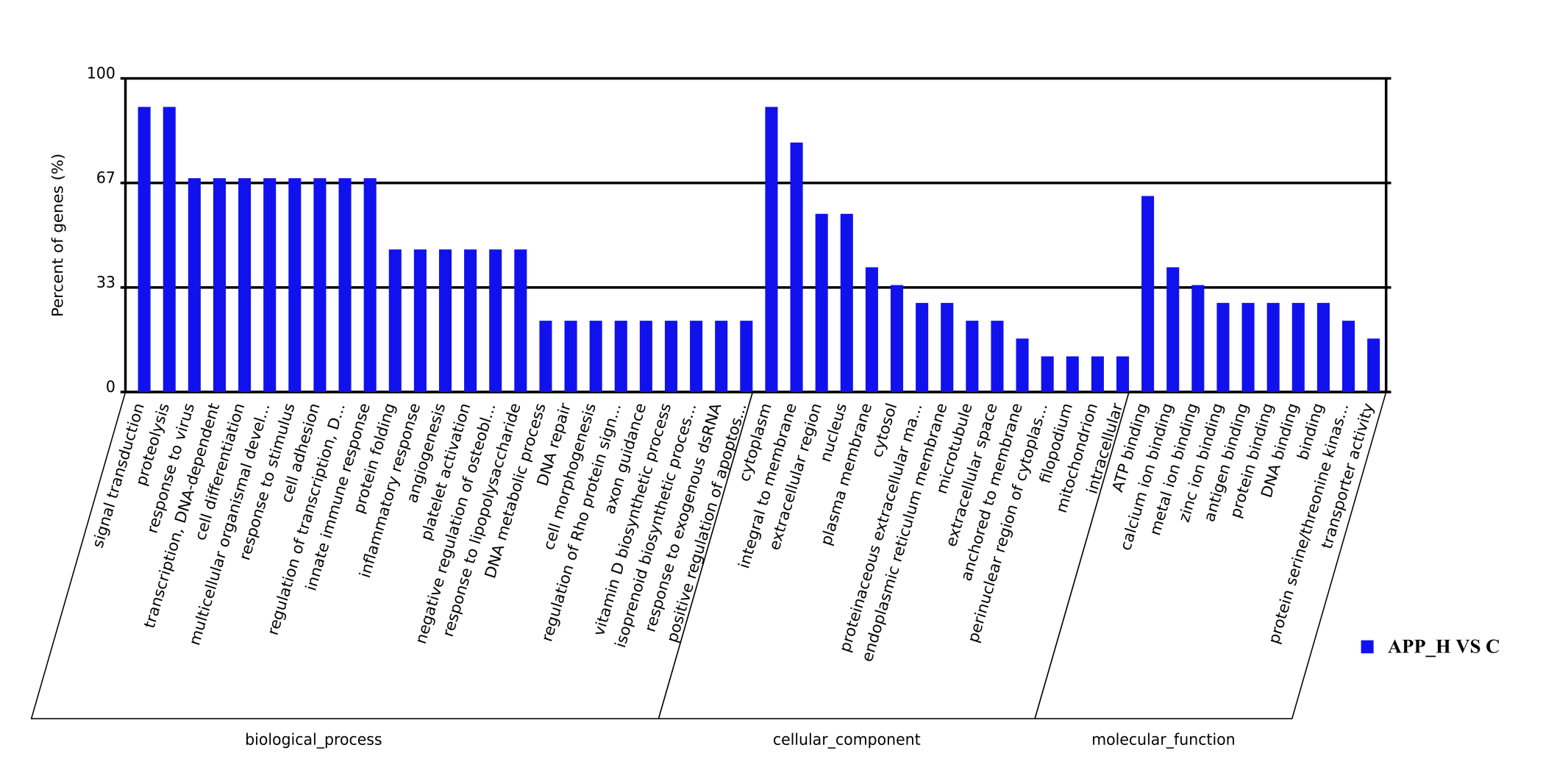


Figure S1. GO function annotation of the differentially expressed genes (corrected *P*-value<0.05) in APP_A VS C and APP_H VS C groups comparisons.
