# Supplementary material for: Effects of dietary supplementation with apple peel powder on the growth, blood and liver parameters, and transcriptome of genetically improved farmed tilapia (GIFT, Oreochromis niloticus)
Source: PLoS One. 2019 Nov 12;14(11):e0224995. doi: 10.1371/journal.pone.0224995 (PMC6850550; doi:10.1371/journal.pone.0224995)
Supplement: S1 Table — (DOCX) [file pone.0224995.s002.docx]

Effects of dietary supplementation with apple peel powder on the growth, blood and liver parameters, and transcriptome of genetically improved farmed tilapia (GIFT, *Oreochromis niloticus*)

Jun Qiang^1*^, Omyia Ahmed Mohamed Khamis^1^, Huo Jin Jiang^2^, Zhe Ming Cao^1^, Jie He^1^, Yi Fan Tao^1^, Pao Xu^1*^

Jin Wen Bao^1^

1. *Key Laboratory of Freshwater Fisheries and Germplasm Resources Utilization, Ministry ofAgriculture, Freshwater Fisheries Research Center, Chinese Academy of Fishery Sciences, Wuxi214081, Jiangsu, China*

2. *Beijing Yujing Biotechnology Co., Ltd.,Beijing 101100, China*

**Additional file 1: S1 Table.** Summary of read data aligned with *Oreochromis_niloticus* transcriptome

| Sample | APP_A1 | APP_A2 | APP_A3 | APP_C1 | APP_C2 |
| --- | --- | --- | --- | --- | --- |
| Valid reads | 45374664 | 57040408 | 40430496 | 48844226 | 53512740 |
| Mapped reads | 27857982(61.40%) | 35474059(62.19%) | 24377649(60.30%) | 31015020(63.50%) | 32605882(60.93%) |
| Unique Mapped reads | 27223513(60.00%) | 34783921(60.98%) | 23814120(45.80%) | 30365348(62.17%) | 31903332(59.62%) |
| Multi Mapped reads | 634469(1.40%) | 690138(1.21%) | 563529(1.08%) | 649672(1.33%) | 702550(1.31%) |
| PE Mapped reads | 10931749(24.09%) | 14032424(24.60%) | 9642941(18.55%) | 12213331(25.00%) | 12576911(23.50%) |
| Mapped left reads | 14957954(32.97%) | 19045619(33.39%) | 13081453(25.16%) | 16695113(34.18%) | 17683208(33.04%) |
| Mapped right reads | 12900028(28.43%) | 16428440(28.80%) | 11296196(21.73%) | 14319907(29.32%) | 14922674(27.89%) |
| Reads map to sense strand | 13688640(30.17%) | 17517465(30.71%) | 11989511(23.06%) | 15337127(31.40%) | 16140229(30.16%) |
| Reads map to antisense strand | 13534873(29.83%) | 17266456(30.27%) | 11824609(22.74%) | 15028221(30.77%) | 15763103(29.46%) |
| Non-splice reads | 11741046(25.88%) | 14866380(26.06%) | 9300128(17.89%) | 12984919(26.58%) | 14102778(26.35%) |
| Splice reads | 15482467(34.12%) | 19917541(34.92%) | 14513992(27.91%) | 17380429(35.58%) | 17800554(33.26%) |

| Sample | APP_C3 | APP_H1 | APP_H2 | APP_H3 |
| --- | --- | --- | --- | --- |
| Valid reads | 49111518 | 47660140 | 62293208 | 51294450 |
| Mapped reads | 29847539(60.78%) | 31551102(66.20%) | 38937856(62.51%) | 31822452(62.04%) |
| Unique Mapped reads | 29262462(59.58%) | 30945936(57.28%) | 38120532(61.20%) | 31473474(61.36%) |
| Multi Mapped reads | 585077(1.19%) | 605166(1.12%) | 817324(1.31%) | 348978(0.68%) |
| PE Mapped reads | 11595784(23.61%) | 11570525(21.42%) | 15729328(25.25%) | 13222368(25.78%) |
| Mapped left reads | 16099610(32.78%) | 18061234(33.43%) | 20622214(33.11%) | 16848422(32.85%) |
| Mapped right reads | 13747929(27.99%) | 13489868(24.97%) | 18315642(29.40%) | 14974030(29.19%) |
| Reads map to sense strand | 14772849(30.08%) | 15774297(29.20%) | 19143089(30.73%) | 15737646(30.68%) |
| Reads map to antisense strand | 14489613(29.50%) | 15171639(28.08%) | 18977443(30.46%) | 15735828(30.68%) |
| Non-splice reads | 12744116(25.95%) | 12684789(23.48%) | 17449844(28.01%) | 13476627(26.27%) |
| Splice reads | 16518346(33.63%) | 18261147(33.80%) | 20670688(33.18%) | 17996847(35.09%) |
