# Supplementary material for: Effects of dietary supplementation with apple peel powder on the growth, blood and liver parameters, and transcriptome of genetically improved farmed tilapia (GIFT, Oreochromis niloticus)
Source: PLoS One. 2019 Nov 12;14(11):e0224995. doi: 10.1371/journal.pone.0224995 (PMC6850550; doi:10.1371/journal.pone.0224995)
Supplement: S2 Table — (DOCX) [file pone.0224995.s003.docx]

**Additional file 1: S2 Table.** Exonic rates (%) of APP_A, APP_H and C libraries regarding the mRNA-seq experiments

| Samples | APP_A1 | APP_A2 | APP_A3 | APP_C1 | APP_C2 | APP_C3 | APP_H1 | APP_H2 | APP_H3 |
| --- | --- | --- | --- | --- | --- | --- | --- | --- | --- |
| exon | 89.2 | 90.04 | 90.84 | 90.03 | 90.02 | 89.91 | 90.49 | 88.65 | 91.38 |
| intron | 4.1 | 3.66 | 3.42 | 3.64 | 3.61 | 3.78 | 3.37 | 4.06 | 2.87 |
| intergenic | 6.7 | 6.3 | 5.75 | 6.33 | 6.36 | 6.32 | 6.14 | 7.3 | 5.75 |
